# Synthesis and Comprehensive Characterization of Bio-Based Heat-Resistant Poly(pentamethylene/dodecamethylene tere phthalamide) Copolymers

**DOI:** 10.3390/polym18182286

**Published:** 2026-09-19

**Authors:** Bingxiao Liu, Haiyu Tian, Binquan Wang, Liqun Ma, Hengjing Luo, Yanye Li, Xingyue Zhang, Chen Yang, Zhongqiang Wang, Mingzheng Hao, Yunzhen Zhu

**Affiliations:** 1 Department of Materials Engineering, Taiyuan Institute of Technology, Taiyuan 030008, China; 18003466744@163.com (H.T.); 18659575326@163.com (B.W.); 18083231409@163.com (H.L.); xingyue202608@163.com (X.Z.); zhuyunzhen0335@163.com (Y.Z.); 2 Shanxi Center of Technology Innovation for Polyamide Materials, Taiyuan 030008, China; 3 Technology Center, Guangdong Sinoplast Advanced Material Co., Ltd., Dongguan 523879, China; lqma0827@163.com (L.M.);; 4 Research and Development Center, Cathay Biotech Inc., Shanghai 201114, China

**Keywords:** bio-based polymers, renewable feedstocks, thermal resistance, thermal degradation, thermogravimetric analysis, activation energy

## Abstract

In this study, bio-based poly(pentamethylene terephthalamide) (PA5T), poly(pentamethy lene/dodecamethylene terephthalamide) (PA5T/12T), and poly(dodecamethylene terephthalamide) (PA12T) were synthesized using 1,5-pentanediamine, 1,12-dodecanediamine and terephthalic acid as polymerization monomers. PA12T segments with longer methylene sequences were incorporated to address the processing challenge of PA5T, whose melting temperature is close to its thermal decomposition temperature. The effects of PA12T segment content on the chemical structure, thermal properties, crystallization behavior and solvent resistance of the copolymers were investigated. Meanwhile, the thermal degradation mechanism was elucidated by thermal degradation kinetics analysis. The results show that PA5T is dominated by the γ-form, whereas PA5T/12T and PA12T exhibit the coexistence of both α- and γ-forms. With increasing content of the PA12T segment, the melting temperature and melting enthalpy of PA5T/12T decrease initially and then increase gradually, which is attributed to the combined effects of longer PA12T segments on the molecular chain architecture and crystallization behavior of the copolymers. PA5T/12T-6:4 and PA12T exhibit melting temperatures of 293.1 °C and 285.6 °C, respectively (both ≥280 °C), which meet the service-temperature requirements for heat-resistant polyamides. Meanwhile, they possess excellent solvent resistance and a wide processing window, showing considerable application potential. The thermal degradation mechanism of both PA5T/12T-6:4 and PA12T follows the R2 model, i.e., the phase-boundary-controlled reaction mechanism (contracting area). The findings provide new strategies for developing bio-based heat-resistant polyamides and deepen our understanding of the thermal degradation mechanisms of this class of polymers.

## 1. Introduction

As one of the most widely used synthetic materials worldwide, plastics have seen a continuous rise in production alongside industrial development, with global annual plastic production being projected to reach 1.6 billion tonnes by 2050, and they have been widely used in various industries and daily life [1,2,3]. Currently, the vast majority of plastics are synthesized using petroleum- and natural gas-derived monomers, and the accelerated depletion of fossil resources has raised increasingly severe resource and environmental concerns [4,5,6,7]. In contrast, bio-based plastics sourced from renewable biomass account for only 1% of the global plastics market [8,9].

Semi-aromatic heat-resistant polyamides, a critical category of high-performance engineering plastics, are often synthesized via the polycondensation of aliphatic diamines with aromatic dicarboxylic acids or aromatic diamines with aliphatic dicarboxylic acids [10,11]. Owing to their distinctive molecular chain architecture, these polymers possess some advantages, such as high melting points, excellent chemical resistance, and superior mechanical strength and rigidity, rendering them irreplaceable in high-end manufacturing sectors, such as electronics/electrical engineering and automotive lightweighting (surface mount devices, LED reflectors, automotive powertrains, etc.) [12,13,14,15]. However, semi-aromatic poly(hexamethylene terephthalamide) (PA6T), which possesses excellent heat resistance, moderate cost and relatively high market share, is a petroleum-based polyamide [5]. Therefore, developing bio-based semi-aromatic polyamides with renewable feedstocks has emerged as a pivotal trend.

1,5-Pentanediamine is structurally similar to 1,6-hexanediamine, which can be produced by microbial transformation of corn starch [16,17,18,19]. At present, there are few reports on semi-aromatic poly(pentamethylene terephthalamide) (PA5T) synthesized from bio-based 1,5-pentanediamine as the main raw material. To address the processing drawbacks of PA5T, Meng et al. copolymerized it with aliphatic poly(pentamethylene adipamide) (PA56) to tune its processing properties, with particular focus on rheological characterization [10]. Li et al. prepared bio-based copolyamide PA5T/54 via solid-state polymerization using nylon salt composed of 1,5-diaminopentane, succinic acid and terephthalic acid as raw materials, and the resulting polymer exhibited better comprehensive properties than that obtained by melt polymerization [20]. Most existing literature focuses on introducing low-cost short-chain diacids such as succinic acid and adipic acid into the PA5T molecular chain to broaden its processing window. However, short-chain aliphatic diacids are prone to intramolecular cyclization during high-temperature polymerization or processing, which degrades the polymer properties. Furthermore, few studies have reported the thermal degradation behavior of its copolymer systems.

1,12-dodecanediamine, another long-chain aliphatic diamine, can be derived from dodecanedioic acid, which is synthesized via microbial fermentation of n-dodecane [21]. Compared with conventional petrochemical synthetic routes, the bio-based synthetic pathway can remarkably reduce the carbon footprint and effectively mitigate the environmental burden.

On the basis of the above background, PA5T was synthesized via a “salt formation–polycondensation” method using 1,5-pentanediamine and terephthalic acid as the main monomers. To address the technical challenge arising from its extremely narrow processing window, varying loadings of long-chain 1,12-dodecanediamine were incorporated into its main molecular chain to synthesize the copolymers, i.e., poly(pentamethylene/dodecamethylene terephthalamide) (PA5T/12T). The chemical structures of PA5T, PA5T/12T, and PA12T were characterized using Fourier transform infrared spectroscopy (FTIR) and ^13^C nuclear magnetic resonance spectroscopy (^13^C-NMR). Their thermal properties and crystallization behavior were systematically investigated using differential scanning calorimetry (DSC), thermogravimetric analysis (TGA), and X-ray diffraction (XRD), and solvent resistance tests were also performed.

As a core means to reveal the thermal stability mechanism of polymers and regulate their thermal processing behavior, thermal degradation kinetics possesses important scientific value and engineering guiding significance for the engineering-scale preparation and thermochemical resource utilization of polymers [22,23,24]. We therefore employed three model-free methods, including Kissinger–Akahira–Sunose (KAS), Flynn–Wall–Ozawa (FWO), and Tang methods, to calculate the thermal degradation activation energies of PA5T/12T-6:4 and PA12T, which feature excellent heat resistance and a relatively wide processing window. Meanwhile, the Coats–Redfern method was used to confirm their thermal degradation mechanisms.

## 2. Materials and Methods

### 2.1. Materials

1,5-pentanediamine was obtained from Cathay Biotech (Shanghai, China). 1,12-dodecanediamine was supplied by Wuxi Yinda Nylon (Wuxi, China). Terephthalic acid was purchased from Aladdin Bio-Chem Technology (Shanghai, China). Deuterated trifluoroacetic acid (TFA-d) was purchased from Shanghai Macklin Biochemical Technology (Shanghai, China). Deionized water was prepared freshly using an OSJ-PLUS-DI-250L ultrapure water system (Jinan OLABO Technology, Jinan, China). Various organic solvents, including anhydrous methanol, tetrahydrofuran (THF), and dimethyl sulfoxide (DMSO), were purchased from Tianjin Beichen Fangzheng Reagent (Tianjin, China). Dimethylformamide (DMF) and concentrated sulfuric acid were provided by Tianjin Tianli Chemical Reagent (Tianjin, China) and Nantong Ruixiang New Materials (Nantong, China), respectively. All reagents were of analytical grade or higher and used without further purification.

### 2.2. Preparation of 5T, 5T/12T, and 12T Salts

As shown in Table 1, 5T, 5T/12T, and 12T salts were prepared using 1,5-pentanediamine, 1,12-dodecanediamine, and terephthalic acid at different molar ratios. They were sequentially added into a three-necked flask equipped with a mechanical stirrer(IKA stirrer, IKA-Werke GmbH & Co. KG, Staufen, Germany), and the reaction was carried out with stirring at above 75 °C for 2 h. After the reaction, the products were dried in a vacuum oven (Shanghai Yiheng Instruments Co., Ltd., Shanghai, China) to obtain 5T, 5T/12T, and 12T salts.

### 2.3. Preparation of PA5T, PA5T/12T, and PA12T

The 5T, 5T/12T, and 12T salts were separately transferred into a high-temperature high-pressure autoclave (Weihai Xingyu Chemical Machinery Co., Ltd., Weihai, China), followed by the addition of deionized water (200 mL). The air inside the autoclave was purged with nitrogen three times. The preparation was performed in two steps. First, the reaction was carried out at 230 °C and an internal pressure below 2.0 MPa for 1 h. Subsequently, the pressure was slowly released to atmospheric pressure over 1.5 h, followed by a vacuum. The temperature was raised to 260 °C, at which the reaction was continued for 3 h to obtain PA5T, PA5T/12T, and PA12T.

### 2.4. Characterization

#### 2.4.1. Fourier Transform Infrared Spectroscopy (FTIR)

Small amounts of 5T, 5T/12T, and 12T salts, as well as PA5T, PA5T/12T, and PA12T powders, were separately ground thoroughly with potassium bromide in an agate mortar(Shanghai Xinnuo Instrument Group Co., Ltd., Shanghai, China), followed by compression into pellets for analysis. FTIR spectra were recorded on a Thermo Nicolet iS50 spectrometer (Thermo Fisher Scientific, Waltham, MA, USA) in transmission mode over a wavenumber range of 4000–500 cm^−1^.

#### 2.4.2. ^13^C Nuclear Magnetic Resonance Spectroscopy (^13^C-NMR)

Appropriate amounts of PA5T, PA5T/12T, and PA12T were separately dissolved in TFA-d in NMR tubes. ^13^C-NMR spectra were recorded on a Bruker DPX-400 spectrometer (Bruker Corporation, Billerica, MA, USA) at 400 MHz.

#### 2.4.3. Differential Scanning Calorimetry (DSC)

Appropriate amounts of PA5T, PA5T/12T, and PA12T were sealed in aluminum pans (Shanghai Zhengji Scientific Instrument Co., Ltd., Shanghai, China), and DSC measurements were performed on a TA Q20 DSC under a nitrogen atmosphere. First, the samples were heated to a temperature above their melting points at a heating rate of 40 °C/min and maintained isothermally for 3 min to erase the thermal history. Then, the samples were cooled to 30 °C at a rate of 20 °C/min and maintained at this temperature for 3 min. Finally, a second heating scan was conducted at 20 °C/min to a temperature above the melting point, during which the DSC thermograms were recorded.

#### 2.4.4. X-Ray Diffraction (XRD)

PA5T, PA5T/12T, and PA12T samples were ground into fine powders prior to analysis. Their crystalline phases were identified using XRD on a Rigaku SmartLab diffractometer (Rigaku, Tokyo, Japan) over a 2θ range of 2–50° at a scanning rate of 2°/min.

#### 2.4.5. Thermogravimetric Analysis (TGA)

Appropriate amounts of PA5T/12T with varying content of PA12T segments were separately placed in crucibles. TGA was performed under a nitrogen atmosphere, heating from room temperature to 700 °C at a heating rate of 10 °C/min, and TG curves were recorded. Moreover, to analyze the thermal degradation kinetics, PA5T/12T with the optimal molar ratio of 6:4 (i.e., PA5T/12T-6:4) and PA12T were heated from room temperature to 700 °C at heating rates of 5, 10, 20, and 30 °C/min, respectively, and their TG curves were recorded.

#### 2.4.6. Solvent Resistance Test

Appropriate amounts of PA5T, PA5T/12T, and PA12T were separately immersed in DMSO, DMF, THF, anhydrous methanol and concentrated sulfuric acid at room temperature. After 48 h of immersion, the dissolution behavior of each sample was observed and recorded.

## 3. Results

### 3.1. FTIR Analysis of PA5T, PA5T/12T, and PA12T

**Figure 1:**
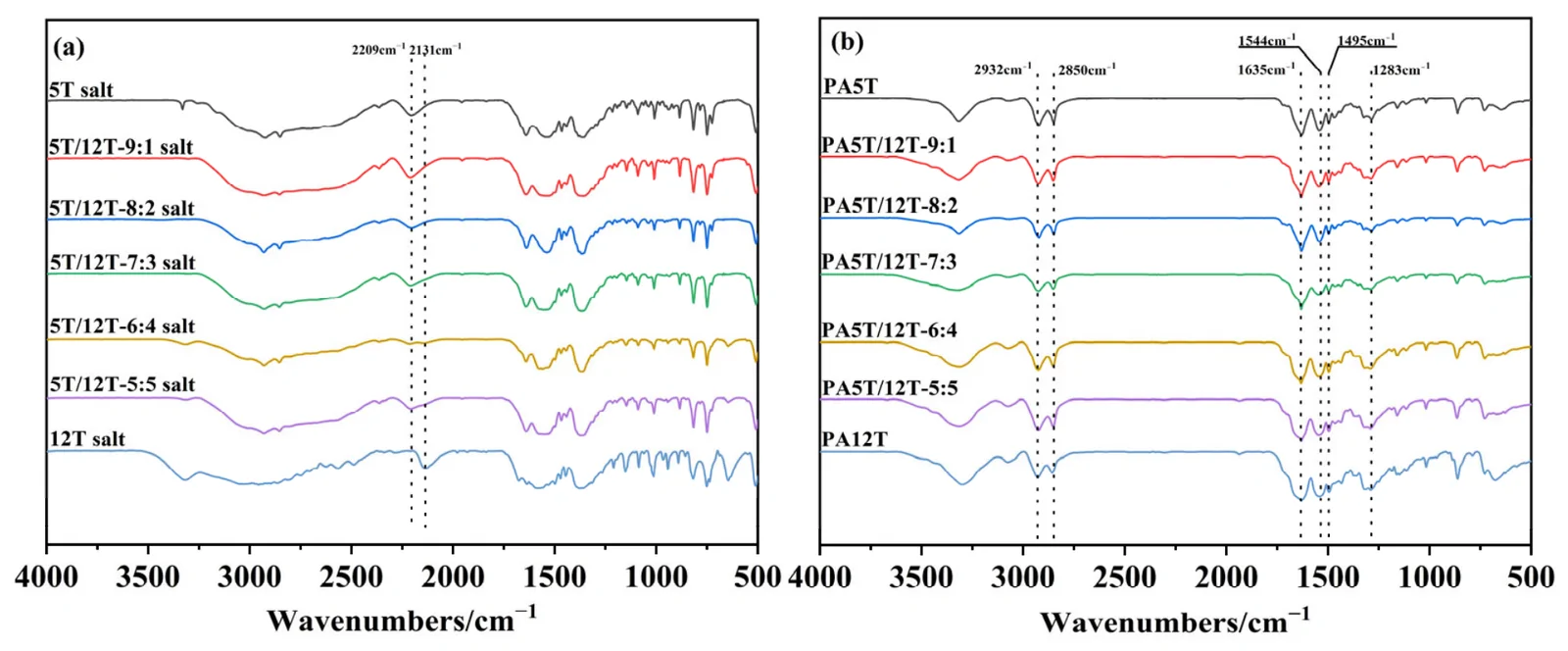
FTIR spectra of (**a**) 5T, 5T/12T, and 12T salts and (**b**) PA5T, PA5T/12T, and PA12T.

In the FTIR spectra of 5T, 5T/12T, and 12T salts (Figure 1a), a broad and intense absorption band attributed to –NH_3_^+^ is observed in the range of 2400–3200 cm^−1^, along with the characteristic overtone absorption peaks of –NH_3_^+^ at 2209 cm^−1^ and 2131 cm^−1^. The peaks between 1536 and 1578 cm^−1^ are assigned to the characteristic stretching vibrations of -COO^–^. The peak positions above collectively confirm the typical structural features of ionic salts. In contrast, there are several new peaks found in the FTIR spectra of PA5T, PA5T/12T, and PA12T (Figure 1b). The absorption peaks at 2932 and 2850 cm^−1^ are assigned to the C–N stretching vibration and the C–H stretching vibration, respectively. The strong absorption peak at 1635 cm^−1^ corresponds to the characteristic amide I band, which is primarily attributed to the C=O stretching vibration of amide groups [25]. The absorption peak at 1544 cm^−1^ is assigned to the characteristic amide II band, which is attributed to the coupling effect of the N–H in-plane bending vibration and the C–N stretching vibration. The characteristic absorption peaks at 1495 cm^−1^ and 1283 cm^−1^ correspond to amide III and amide IV, respectively, which arise from the C–N stretching vibration and the C–CO stretching vibration, respectively [26,27]. Notably, the overtone absorption band of –NH_3_^+^ present in the spectra of salts completely disappears in the spectra of copolymers, while a series of four characteristic amide bands (I, II, III, and IV) emerge clearly, indicating the successful synthesis of PA5T, PA5T/12T, and PA12T.

### 3.2. ^13^C-NMR Analysis of PA5T, PA5T/12T, and PA12T

**Figure 2:**
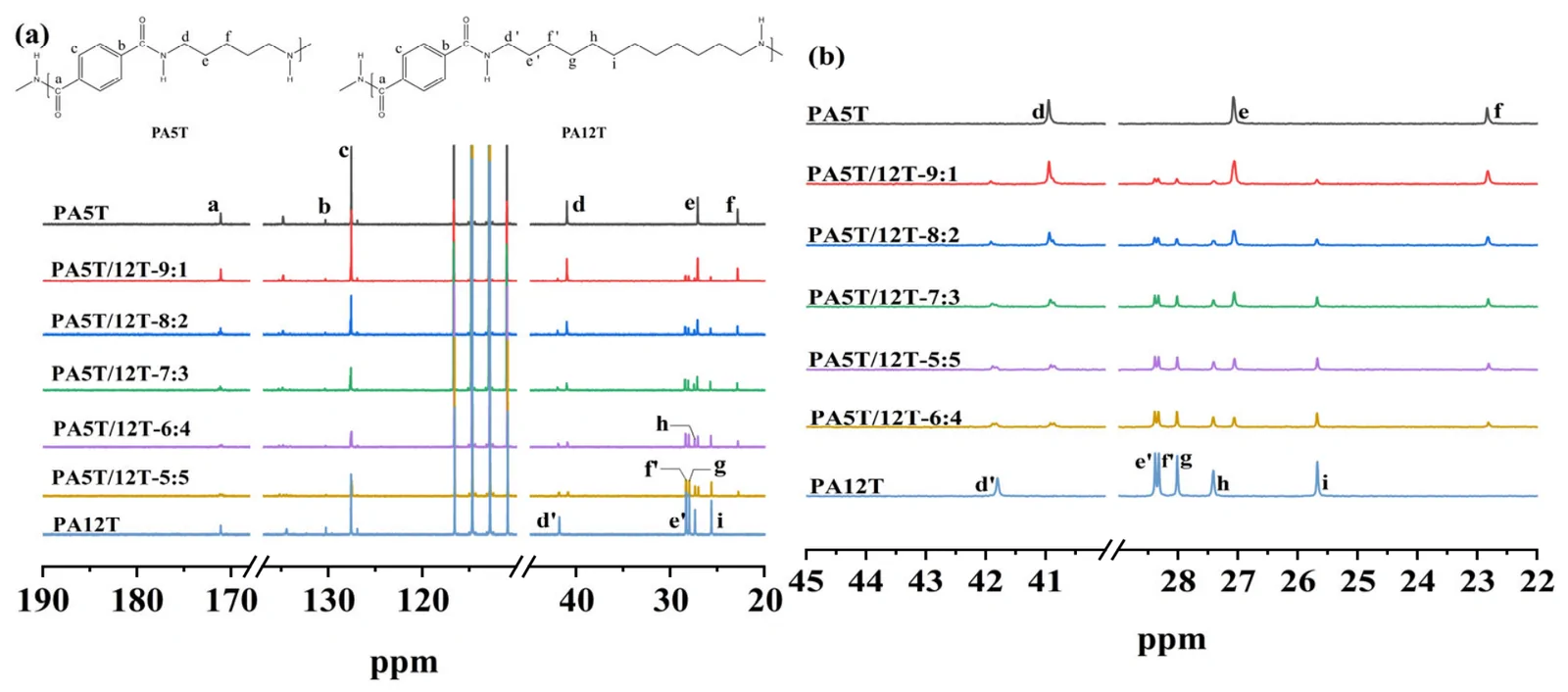
^13^C-NMR spectra of PA5T, PA5T/12T, and PA12T: (**a**) full region and (**b**) expanded region.

The ^13^C-NMR spectra of PA5T, PA5T/12T, and PA12T are presented in Figure 2a. The peak at 171.19 ppm is assigned to carbonyl carbon atoms (position a). The peak at 134.83 ppm is attributed to carbon atoms of the benzene ring bonded to the carbonyl group (position b), while the peak at 127.51 ppm corresponds to the ortho-carbon atoms of the benzene ring (position c) [28]. The presence of these benzene ring-related characteristic peaks confirms the existence of benzene ring units in the molecular structures of PA5T, PA5T/12T, and PA12T. The characteristic peaks of methylene carbons adjacent to the amide bonds exhibit distinctly different positions among these polymers (Figure 2a,b). The characteristic peak of the α-methylene carbon adjacent to the amide bond in PA5T appears at 40.95 ppm (position d), whereas the corresponding peak in PA12T is observed at 41.81 ppm (position d′). The β- and γ-methylene carbons adjacent to the amide bond in PA5T correspond to the characteristic peaks at 27.08 ppm (position e) and 22.84 ppm (position f), respectively. In contrast, the corresponding peaks in PA12T appear at 28.39 ppm (position e′) and 28.32 ppm (position f′), respectively.

Furthermore, due to its longer carbon chain, PA12T exhibits additional characteristic peaks at 28.01 ppm (position g), 27.41 ppm (position h), and 25.68 ppm (position i), which are assigned to the δ-, ε-, and i-methylene carbons, respectively. These peaks are fully consistent with the molecular structural characteristics of PA12T. It is noteworthy that, in the expanded spectra at positions d, e, and f that correspond to the α-, β-, and γ-methylene carbons of PA5T, respectively (Figure 2b), the peak intensities at these positions decrease gradually and eventually disappear with increasing content of PA12T segments. Concurrently, the peak intensities at positions d′, e′, f′, g, h, and i (corresponding to the α-, β-, γ-, δ-, ε-, and i-methylene carbons of PA12T, respectively) increase gradually.

To verify the actual composition of the copolymers, quantitative analysis was performed using the α-methylene peaks (peak d for PA5T and peak d′ for PA12T) as the reference. Since the repeating units of PA5T and PA12T contain an equal number of amide-adjacent α-methylene carbon atoms, the molar ratio of PA5T segments to PA12T segments can be calculated from the integral ratio of peak d to peak d′. The results are summarized in Table 2, revealing that the actual compositions of the copolymers are generally consistent with the feed molar ratios. All the above spectral assignments agree well with the theoretical chemical shifts in PA5T, PA5T/12T and PA12T. Combined with the quantitative composition results, it further confirms that the as-synthesized copolymers possess the expected molecular structures.

### 3.3. Melting Temperatures and Enthalpies of PA5T, PA5T/12T, and PA12T

**Figure 3:**
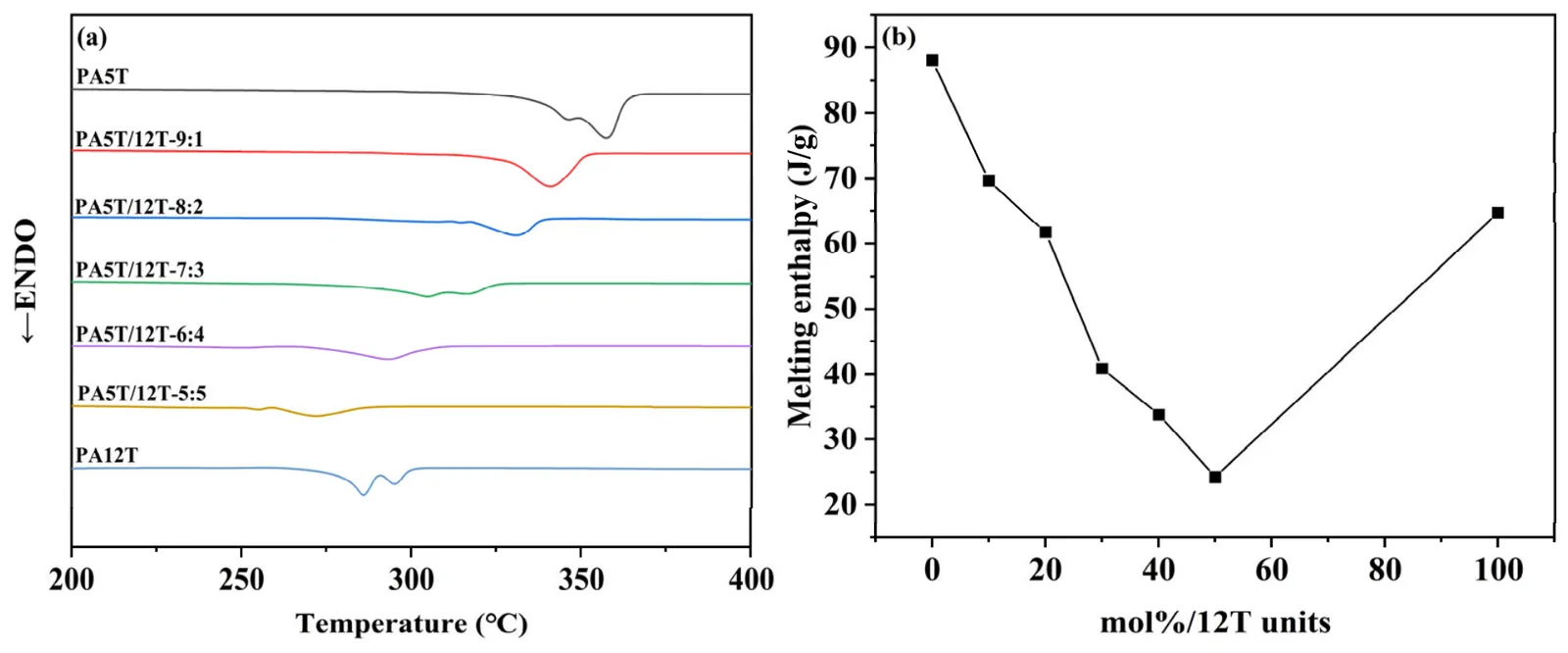
DSC curves (**a**) and melting enthalpy (**b**) of PA5T, PA5T/12T, and PA12T.

DSC analysis shows that the melting temperature of PA5T is 357.79 °C (Figure 3a), which is significantly higher than that of PA12T (285.86 °C), possibly due to the fact that, compared with PA12T, PA5T possesses shorter molecular chains with a higher density of rigid benzene rings within the segments, along with stronger interchain interactions. In addition, with increasing content of PA12T segments, the melting enthalpy of the copolymers decreases gradually (Figure 3b), which suggests that the incorporation of PA12T segments disrupts the molecular chain regularity of PA5T and suppresses the crystallization ability of the copolymers. Meanwhile, the longer molecular chains and higher segmental flexibility of PA12T further reduce the chain rigidity of the copolymers, resulting in a gradual decrease in the melting temperature. In contrast, pure PA12T exhibits high molecular chain regularity and favorable crystallization capacity, which corresponds to a larger melting enthalpy (Figure 3b). Furthermore, double melting peaks are observed in the curves of certain samples, which can be attributed to the recrystallization behavior during the crystallization and melting processes of the polymers [10].

### 3.4. Crystalline Structures of PA5T, PA5T/12T, and PA12T

**Figure 4:**
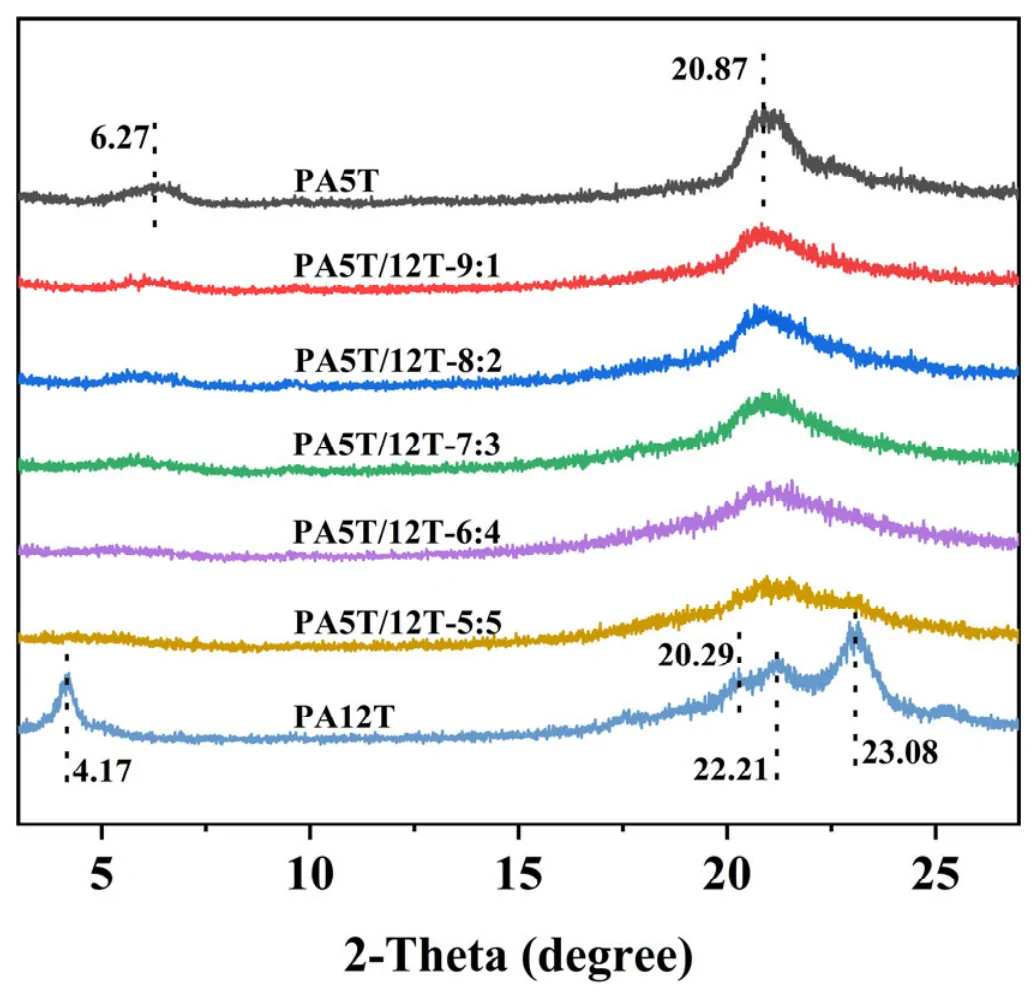
XRD patterns of PA5T, PA5T/12T, and PA12T.

**Figure 5:**
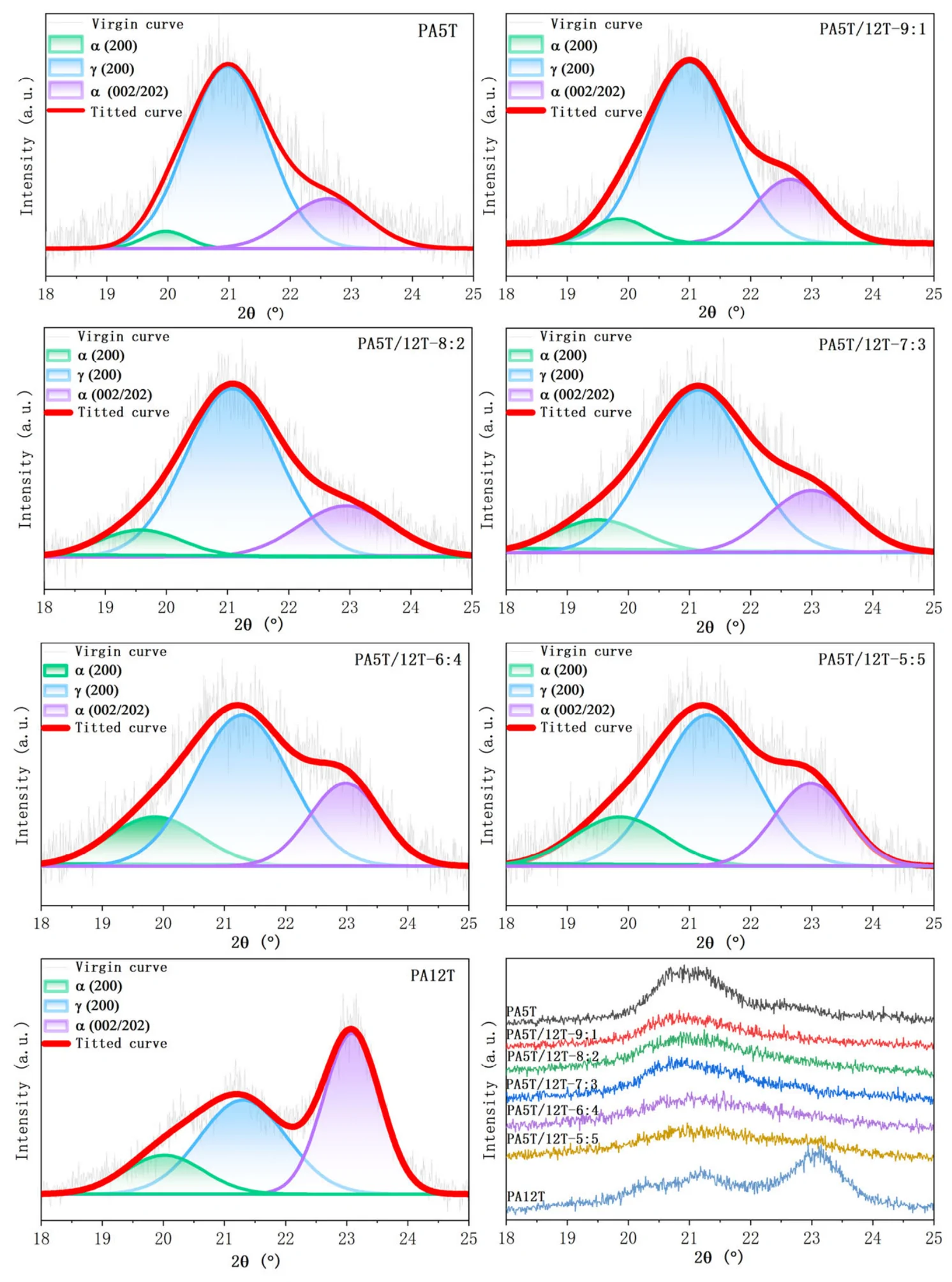
XRD peak deconvolution profiles of PA5T, PA5T/12T and PA12T.

Figure 4 presents the XRD patterns of PA5T, PA5T/12T, and PA12T. Specifically, the diffraction peak of PA5T at 2θ = 6.27° corresponds to the diffraction signal of the (001) crystal plane, which is primarily attributed to the ordered arrangement of saturated aliphatic segments within the molecular chains [29]. The diffraction peak at 2θ = 20.87° further indicates that the main chain of PA5T adopts a γ-crystal form [30]. PA12T exhibits a long-period diffraction peak of the (001) crystal plane at 2θ = 4.17°, which originates from the ordered lamellar stacking structure formed by the longer dodecamethylene segments within its molecular chains. There are three characteristic diffraction peaks at 2θ = 20.29°, 22.21°, and 23.08°, which correspond to α(200), γ(200) and α(002/202) crystal planes, respectively [30]. To clarify the relative evolution of α- and γ-crystal forms, peak deconvolution analysis was performed on the wide-angle diffraction region of 2θ = 18–25° (Figure 5). The peak positions, integrated areas of deconvoluted peaks, and calculated relative content of γ-crystal form are summarized in Table 3. The semi-quantitative results further confirm that the relative content of γ-crystal form decreases monotonically from 78.13% for neat PA5T to 40.34% for neat PA12T, demonstrating that the introduction of long dodecamethylene segments drives a gradual transition of the crystalline structure of copolyamides from γ-form-dominated to α-form-dominated.

### 3.5. Thermogravimetric Degradation of PA5T, PA5T/12T, and PA12T

**Figure 6:**
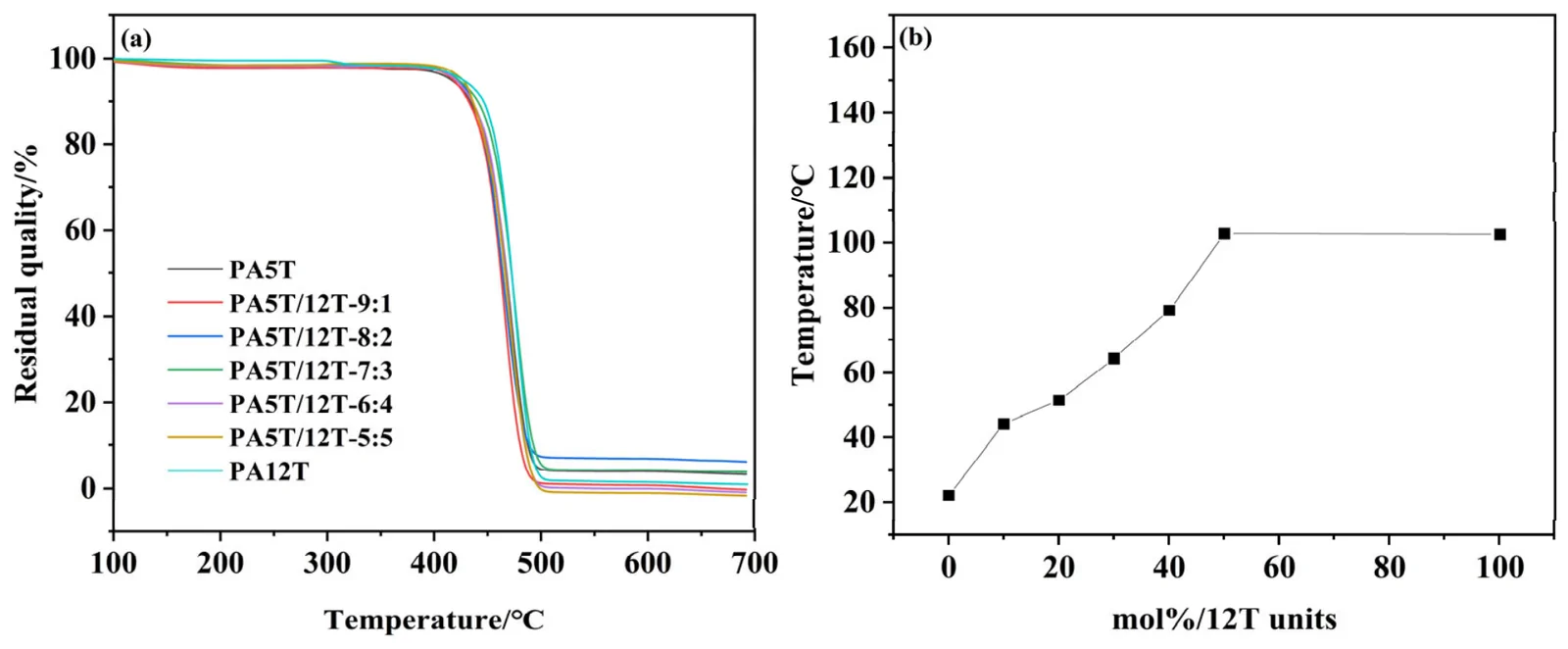
TG curves (**a**) and processing windows (**b**) of PA5T, PA12T and PA5T/12T.

The TG curves of PA5T, PA5T/12T, and PA12T are presented in Figure 6a. All polymers exhibit a single weight-loss stage over the temperature range of 100–500 °C, indicating that all samples undergo one-step degradation, which is consistent with the degradation mechanism of polycondensation polymers. The melting temperature (*T_m_*), extrapolated onset temperature of thermal decomposition (*T_onse_*_t_), and calculated processing window (defined as *T_onse_*_t_ − *T_m_*) for all samples are summarized in Table 4. The variation trend of the processing window of the copolymers with PA12T segment content is shown in Figure 6b. (It should be noted that this calculated parameter alone cannot directly reflect the practical melt-processability of the copolymers.) It can be observed that the processing window of the copolymers broadens with increasing contents of PA12T segments. PA5T/12T-6:4 and PA12T exhibit melting temperatures of 293.1 °C and 285.6 °C, respectively (both ≥ 280 °C), which meet the heat-resistance requirements for high-temperature polyamides. Meanwhile, they possess wide processing windows (79.12 °C for PA5T/12T-6:4 and 102.4 °C for PA12T), showing considerable application potential [31]. Compared with the reported PA5T/54 system, the present series of copolymers deliver a higher melting temperature, onset thermal-decomposition temperature and processing window (the melting temperature of PA5T/54 is 277 °C, and its thermal decomposition temperature is 283 °C) [20].

### 3.6. Thermal Degradation Kinetics of PA5T/12T-6:4 and PA12T

#### 3.6.1. Model-Free Methods

In this study, three model-free methods (KAS, FWO, and Tang), combined with the CR method, were employed to investigate the thermal degradation kinetics of PA5T/12T-6:4 and PA12T, both of which exhibit excellent thermal resistance and relatively wide processing windows.

Generally, for the thermal degradation of solids under non-isothermal conditions, combined with the Arrhenius equation, the following expression can be derived [32]:

(1) $$g\left(\alpha \right)\;=\;\int_{0}^{\alpha }\frac{d_{\alpha }}{\int \left(\alpha \right)}\;=\;\int_{T_{0}}^{T_{P}}\frac{A}{\beta }\exp\left(-\frac{E}{RT}\right)dT\;=\;\frac{AE}{\beta T}P(\chi )$$

where α is the degree of conversion; *A* is the pre-exponential factor; *β* is the heating rate; *T* is the corresponding temperature; *E* is the apparent activation energy; *R* is the gas constant; *χ* is equal to *E*/*RT*.

The KAS method is an integral isoconversional method and assumes that P(*χ*) ≌ *χ*^−2^*e*^−*χ*^. Substituting it into Equation (1) yields [33]:

(2) $$\ln\frac{\beta }{T^{2}}\;=\;\ln\frac{AR}{g(\alpha )E}\;-\;\frac{E}{RT}$$

The FWO method is based on Doyle’s approximation. By substituting it into Equation (1), the following equation can be derived [34]:

(3) $$\ln\beta \;=\;\ln\frac{AE}{g\left(\alpha \right)R}\;-\;2.315\;-\;\frac{0.4567E}{RT}$$

The Tang method takes the logarithm of both sides of Equation (1) and yields the following expression [35]:

(4) $$\ln\left(\frac{\beta }{T^{1.894661}}\right)\;=\;\ln\left(\frac{AE}{g\left(\alpha \right)R}\right)\;+\;3.635041\;-\;1.894661\ln E\;-\;\frac{1.001450E}{RT}$$

Based on Equations (2)–(4), the plots of ln(*β*/*T*^2^), ln*β*, and ln(*β/T*^1.1894661^) versus 1000/*T* were constructed in Figures S1–S3, respectively. The thermal degradation activation energies of PA5T/12T-6:4 and PA12T at different conversion extents can be calculated from the slopes of fitted lines. The correlation coefficients (*R*^2^) and thermal degradation activation energies calculated by the KAS, FWO, and Tang methods are summarized in Table 5. All the fitted lines exhibit high correlation coefficients (*R*^2^ ≥ 0.98), and the average thermal degradation activation energies of PA5T/12T-6:4 and PA12T calculated by the three methods are in good agreement with each other, indicating that all three methods are suitable for determining the thermal degradation activation energies of PA5T/12T-6:4 and PA12T.

#### 3.6.2. Coats–Redfern Method

When the Coats–Redfern (CR) model-fitting method is adopted to calculate the apparent activation energies corresponding to different mechanism models, the activation energies obtained by model-free isoconversional methods such as Kissinger–Akahira–Sunose (KAS), Flynn–Wall–Ozawa (FWO) and Tang are generally used as the reference benchmark, combined with the correlation coefficient of linear fitting for comprehensive evaluation. The mechanism model that achieves the best agreement in activation energy values with the model-free results and yields a favorable correlation coefficient is regarded as the most probable mechanism model for the target reaction [34].

The CR method requires the prior assumption of a thermal degradation mechanism. By substituting ln(1−2*RT*/*E*) → 0 into Equation (1), the following expression can be obtained [36]:

(5) $$\ln\frac{g(\alpha )}{T^{2}}\;=\;\ln\frac{AR}{\beta E}\;-\;\frac{E}{RT}$$

The *g*(*α*) values corresponding to different mechanism functions are listed in Table S1. The different *g*(*α*) functions were separately substituted into Equation (5), and the temperatures corresponding to different conversion extents at various heating rates were then used to construct the plots of ln[*g*(*α*)/*T*^2^] versus 1000/*T* for PA5T/12T-6:4 and PA12T, respectively. The thermal degradation activation energies for different mechanism functions at various heating rates can be calculated from the slopes of the fitted lines, and the results and correlation coefficients of the fitted lines are listed in Tables S2 and S3, respectively. Notably, the thermal degradation activation energies of PA5T/12T-6:4 calculated by three model-free methods are highly consistent with those calculated by the CR method at a heating rate of 10 °C/min based on the phase-boundary-controlled reaction (contracting area) (R2), accompanied by a high correlation coefficient. This indicates that the thermal degradation process of PA5T/12T-6:4 follows the phase-boundary-controlled degradation mechanism (R2). The similar degradation mechanism (R2) also applies to the thermal degradation process of PA12T.

### 3.7. Solvent Resistance of PA5T, PA5T/12T and PA12T

The solvent resistance of PA5T, PA5T/12T, and PA12T was evaluated in five organic solvents, including DMSO, DMF, THF, anhydrous methanol, and concentrated sulfuric acid. As shown in Table 6, all samples are only soluble in concentrated sulfuric acid; no dissolution or swelling was observed in other solvents, demonstrating excellent solvent resistance (i.e., high chemical stability), possibly due to their dense molecular chain structure with strong intermolecular chain interactions.

## 4. Conclusions

In this study, PA5T, PA5T/12T, and PA12T were successfully synthesized via a “salt formation–polycondensation” approach using bio-based 1,5-diaminopentane, 1,12-dodecanediamine, and terephthalic acid as the principal monomers. The incorporation of PA12T segments with longer methylene sequences effectively overcomes the processing challenge of PA5T due to its inherently narrow processing window. PA5T exhibits a single γ-form, whereas both PA5T/12T and PA12T display the coexistence of α- and γ-crystal forms. With the increase in PA12T segment content, the melting temperature and melting enthalpy of PA5T/12T decrease remarkably. This is attributed to the fact that the introduction of flexible PA12T segments reduces the molecular chain rigidity of the copolymers and suppresses their crystallization ability. The melting temperatures of PA5T/12T-6:4 and PA12T are 293.1 °C and 285.6 °C, respectively, which are higher than 280 °C, meeting the requirements for high-temperature polyamides. PA5T/12T-6:4 and PA12T possess wide processing windows (79.12 °C and 102.4 °C, respectively) and excellent solvent resistance. Furthermore, the thermal degradation of both PA5T/12T-6:4 and PA12T follows the R2 mechanism (phase-boundary-controlled reaction (contracting area)).

## Figures and Tables

**Table 1:** Preparation of PA5T, PA5T/12T, and PA12T salts using 1,5-pentanediamine, 1,12-dodecanediamine, and terephthalic acid at different molar ratios.

Samples	1,5-Pentanediamine/mol	1,12-Dodecanediamine/mol	Terephthalic Acid/mol	Water/wt%
PA5T	1	0	1	30
PA5T/12T-9:1	0.9	0.1	1	30
PA5T/12T-8:2	0.8	0.2	1	30
PA5T/12T-7:3	0.7	0.3	1	30
PA5T/12T-6:4	0.6	0.4	1	30
PA5T/12T-5:5	0.5	0.5	1	30
PA12T	0	1	1	30

**Table 2:** Feed and actual compositions of PA5T/12T copolymers from quantitative ^13^C NMR.

Sample	Feed Molar Ratio (PA5T:PA12T)	Actual Molar Ratio (PA5T:PA12T)	Feed PA5T Fraction (mol%)	Actual PA5T Fraction (mol%)
PA5T/12T-9:1	90:10:00	91.5:8.5	90	91.5
PA5T/12T-8:2	80:20:00	80.8:19.2	80	80.8
PA5T/12T-7:3	70:30:00	68.8:31.2	70	68.8
PA5T/12T-6:4	60:40:00	57.9:42.1	60	57.9
PA5T/12T-5:5	50:50:00	49.6:50.4	50	49.6

**Table 3:** XRD peak deconvolution results and γ-form fraction of PA5T, PA5T/12T and PA12T.

Sample	Peak Position 2θ (°) & Integral Area			γ-Crystallinity (%)
PA5T	20.01 (54.63)	21.01 (1727.93)	22.70 (429.08)	78.13
PA5T/12T-9:1	19.96 (56.52)	20.97 (1094.81)	22.61 (292.72)	75.81
PA5T/12T-8:2	19.84 (128.86)	21.00 (1423.00)	22.64 (423.08)	72.05
PA5T/12T-7:3	19.57 (205.47)	21.08 (1481.16)	22.94 (448.99)	69.35
PA5T/12T-6:4	19.50 (232.84)	21.14 (1330.41)	22.99 (436.63)	66.52
PA5T/12T-5:5	19.86 (354.72)	21.29 (1118.20)	22.98 (468.74)	57.00
PA12T	20.01 (462.58)	21.30 (1251.02)	23.09 (1387.34)	40.34

**Table 4:** Melting temperature (*T_m_*), *T_onset_* and calculated processing window of PA5T, PA5T/12T and PA12T.

Samples	*T_m_*/°C	*T_onset_*/°C	Processing Window/°C
PA5T	358.6	380.78	22.18
PA5T/12T-9:1	341.3	385.49	44.19
PA5T/12T-8:2	330.9	382.35	51.45
PA5T/12T-7:3	315.7	380.02	64.32
PA5T/12T-6:4	293.1	372.22	79.12
PA5T/12T-5:5	271.7	374.41	102.71
PA12T	285.8	388.23	102.43

**Table 5:** Thermal degradation activation energies of PA5T/12T-6:4 and PA12T at different conversion extents calculated by KAS, FWO, and Tang methods.

Conversion Extents (%)	KAS				FWO				Tang			
	PA5T/12T-6:4		PA12T		PA5T/12T-6:4		PA12T		PA5T/12T-6:4		PA12T	
	*E*/kJ·mol^−1^	*R* ^2^	*E*/kJ·mol^−1^	*R* ^2^	*E*/kJ·mol^−1^	*R* ^2^	*E*/kJ·mol^−1^	*R* ^2^	*E*/kJ·mol^−1^	*R* ^2^	*E*/kJ·mol^−1^	*R* ^2^
10	234.10	0.98	277.98	0.99	233.98	0.99	275.72	0.99	234.39	0.98	278.21	0.99
20	237.12	0.98	245.08	0.99	237.05	0.98	244.63	0.99	237.42	0.98	245.36	0.99
30	233.32	0.98	248.03	0.99	233.55	0.99	247.56	0.99	233.63	0.98	248.32	0.99
40	229.01	0.99	250.01	0.99	229.53	0.99	249.51	0.99	229.33	0.99	250.30	0.99
50	235.91	0.99	262.01	0.99	236.17	0.99	260.98	0.99	236.22	0.99	262.27	0.99
60	238.11	0.99	263.54	0.99	238.32	0.99	262.51	0.99	238.42	0.99	263.82	0.99
70	240.43	0.99	256.85	0.99	240.61	0.99	256.19	0.99	240.75	0.99	257.14	0.99
80	242.69	0.99	248.38	0.99	242.81	0.99	248.19	0.99	243.00	0.99	248.69	0.99
90	245.57	0.99	234.55	0.99	245.63	0.99	235.13	0.99	245.88	0.99	234.91	0.99
mean	237.36		254.05		237.51		253.38		237.67		254.33	

**Table 6:** Solvent resistance of PA5T, PA5T/12T and PA12T.

Solvents	PA5T	PA5T/12T-9:1	PA5T/12T-8:2	PA5T/12T-7:3	PA5T/12T-6:4	PA5T/12T-5:5	PA12T
DMSO	-	-	-	-	-	-	-
DMF	-	-	-	-	-	-	-
THF	-	-	-	-	-	-	-
anhydrous methanol	-	-	-	-	-	-	-
concentrated sulfuric acid	+	+	+	+	+	+	+

Dissolution: +; Insoluble: -.

## Data Availability

The original contributions presented in the study are included in the article/supplementary material, further inquiries can be directed to the corresponding authors.

## Supplementary Materials

The following supporting information can be downloaded at: https://www.mdpi.com/article/10.3390/polym18182286/s1, Figure S1: Linear plots for calculating the thermal degradation activation energies of PA5T/12T-6:4 and PA12T using the KAS method; Figure S2: Linear plots for calculating the thermal degradation activation energies of PA5T/12T-6:4 and PA12T using the FWO method; Figure S3: Linear plots for calculating the thermal degradation activation energies of PA5T/12T-6:4 and PA12T using the Tang method; Table S1: Functional expressions of *g*(*α*) for different models; Table S2: Thermal degradation activation energies of PA5T/12T-6:4 calculated by the Coats-Redfern method; Table S3. Thermal degradation activation energies of PA12T calculated by the Coats-Redfern method.
