# Supplementary material for: Synthesis and Comprehensive Characterization of Bio-Based Heat-Resistant Poly(pentamethylene/dodecamethylene tere phthalamide) Copolymers
Source: Polymers (Basel). 2026 Sep 19;18(18):2286. doi: 10.3390/polym18182286 (PMC13611394; doi:10.3390/polym18182286)
Supplement: Supplementary file 1 [file polymers-18-02286-s001.zip › polymers-4531177-supplementary.pdf]

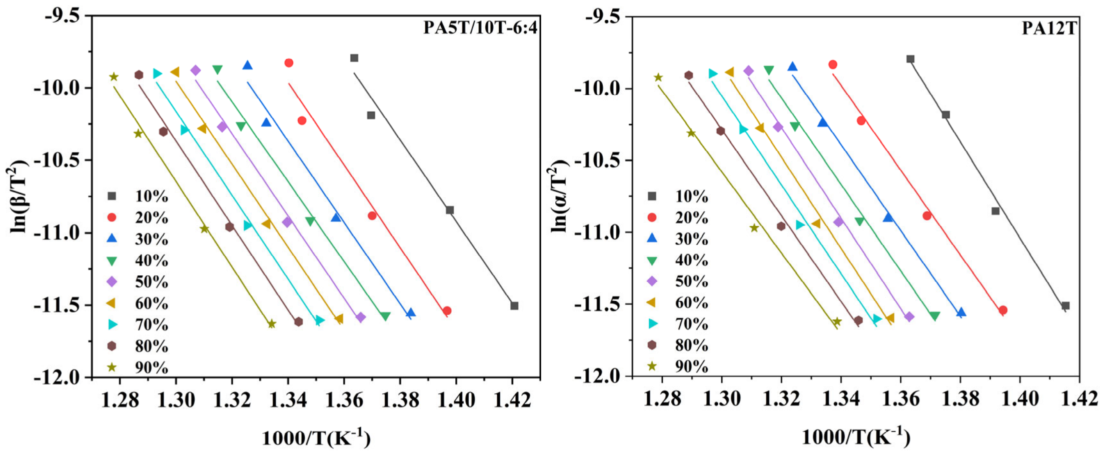

Figure S1. Linear plots for calculating the thermal degradation activation energies of PA5T/12T-6:4 and PA12T using the KAS method

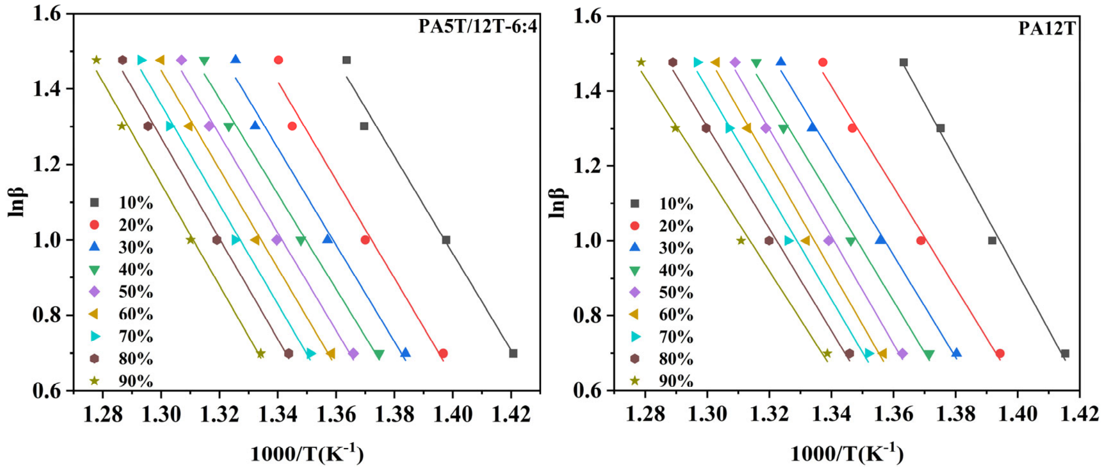

Figure S2. Linear plots for calculating the thermal degradation activation energies of PA5T/12T-6:4 and PA12T using the FWO method

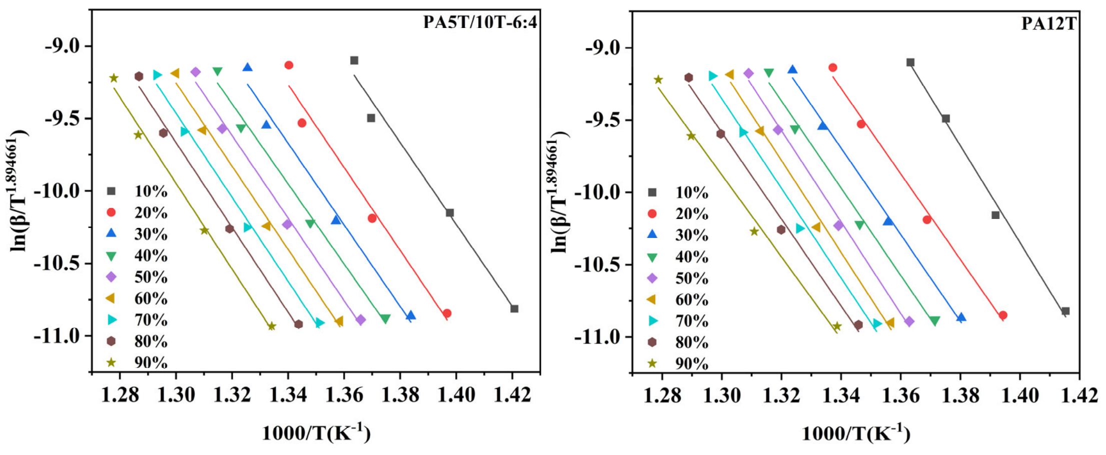

Figure S3. Linear plots for calculating the thermal degradation activation energies of PA5T/12T-6:4 and

PA12T using the Tang method

Table S1. Functional expressions of $g(\alpha)$ for different models

**Solid-state processes**	**Models**	$g(\alpha)$
Sigmoidal Curves		
Nucleation and growth [Avrami-Erofeev,eq. (1)]	A2	$[-\ln(1-\alpha)]^{1/2}$
Nucleation and growth [Avrami-Erofeev,eq. (2)]	A3	$[-\ln(1-\alpha)]^{1/3}$
Nucleation and growth [Avrami-Erofeev,eq. (3)]	A4	$[-\ln(1-\alpha)]^{1/4}$
Deceleration Curves		
One-dimensional diffusion	D1	α^2
Two-dimensional diffusion (Valensi equation)	D2	$(1-\alpha)\ln(1-\alpha)+\alpha$
Three-dimensional diffusion (Jander equation)	D3	$[1-(1-\alpha)^{1/3}]^2$
Three-dimensional diffusion (Ginstling-Brounshtein equation)	D4	$[1-(2/3)\alpha]-(1-\alpha)^{2/3}$
Phase-boundary-controlled reaction (one-dimensional movement)	R1	α
Phase-boundary-controlled reaction (contracting area)	R2	$1-(1-\alpha)^{1/2}$
Phase-boundary-controlled reaction (Spherical symmetric contraction)	R3	$1-(1-\alpha)^{1/3}$

Table S2. Thermal degradation activation energies of PA5T/12T-6:4 calculated by the Coats-Redfern method

	5 °C/min		10 °C/min		20 °C/min		30 °C/min	
	E/kJ·mol^{-1}	R^2	E/kJ·mol^{-1}	R^2	E/kJ·mol^{-1}	R^2	E/kJ·mol^{-1}	R^2
A2	137.58	0.99	136.47	0.99	145.06	0.99	136.80	0.99
A3	87.69	0.99	86.88	0.99	92.53	0.99	87.00	0.99
A4	62.75	0.99	62.09	0.99	66.27	0.99	62.10	0.99
D1	422.18	0.98	422.13	0.99	446.08	0.99	423.21	0.99
D2	467.28	0.99	466.32	0.99	493.24	0.99	467.71	0.99
D3	524.62	0.99	522.31	0.99	553.14	0.99	524.13	0.99
D4	486.13	0.99	484.73	0.99	512.93	0.99	486.26	0.99
R1	205.05	0.98	204.92	0.99	216.78	0.99	205.30	0.99
R2	242.17	0.99	241.23	0.99	255.57	0.99	241.89	0.99
R3	256.27	0.99	255.01	0.99	270.31	0.99	255.77	0.99

Table S3. Thermal degradation activation energies of PA12T calculated by the Coats-Redfern method

	5 ℃/min		10 ℃/min		20 ℃/min		30 ℃/min	
	E/kJ·mol^{-1}	R^2	E/kJ·mol^{-1}	R^2	E/kJ·mol^{-1}	R^2	E/kJ·mol^{-1}	R^2
A2	153.55	0.99	147.84	0.99	143.62	0.98	144.76	0.99
A3	98.34	0.99	94.46	0.99	91.59	0.98	92.31	0.99
A4	70.73	0.99	67.76	0.99	65.57	0.98	66.08	0.99
D1	472.29	0.99	454.52	0.99	443.93	0.99	445.61	0.99
D2	521.38	0.99	502.29	0.99	490.09	0.99	492.56	0.99
D3	583.47	0.99	562.88	0.99	548.67	0.99	552.28	0.99
D4	541.80	0.99	522.21	0.99	509.35	0.99	512.19	0.99
R1	230.11	0.99	221.10	0.99	215.72	0.99	216.50	0.98
R2	270.42	0.99	260.38	0.99	253.68	0.99	255.15	0.99
R3	285.70	0.99	275.29	0.99	268.09	0.99	269.84	0.99
